# Supplementary material for: Repression of Acetaminophen-Induced Hepatotoxicity in HepG2 Cells by Polyphenolic Compounds from Lauridia tetragona (L.f.) R.H. Archer
Source: Molecules. 2019 Jun 4;24(11):2118. doi: 10.3390/molecules24112118 (PMC6600165; doi:10.3390/molecules24112118)

Figure S1: Total ion chromatogram of PPRF1

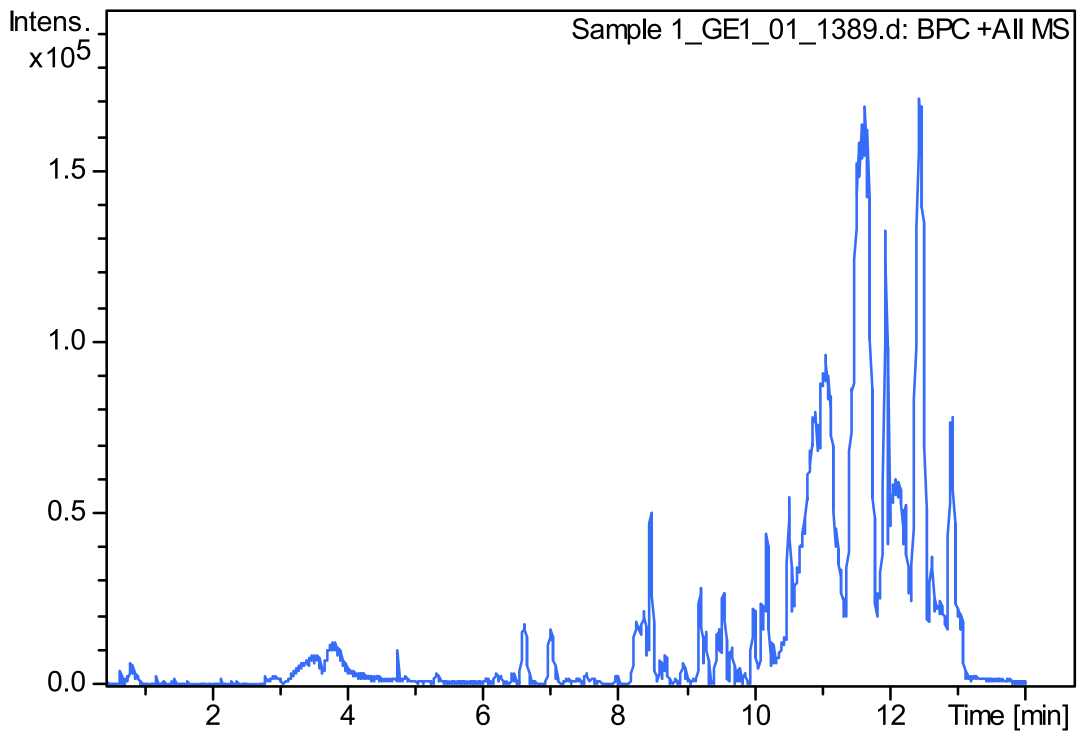

Figure S2: Total ion chromatogram of PPRF2

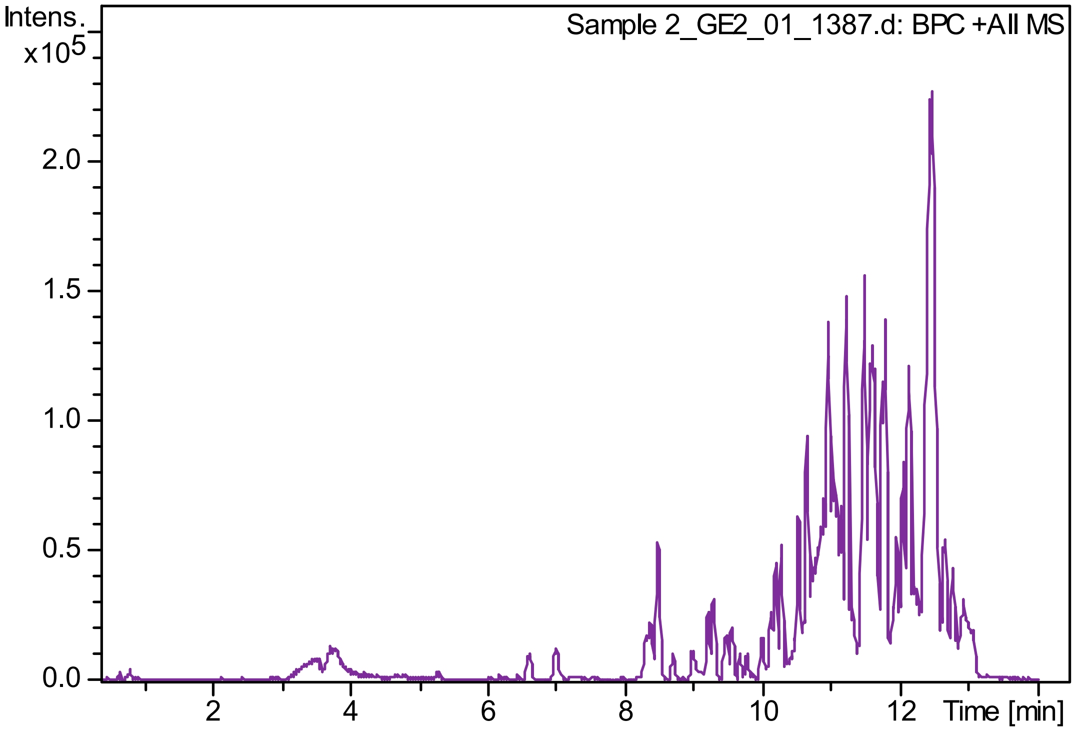

Figure S3: Total ion chromatogram of PPRF3

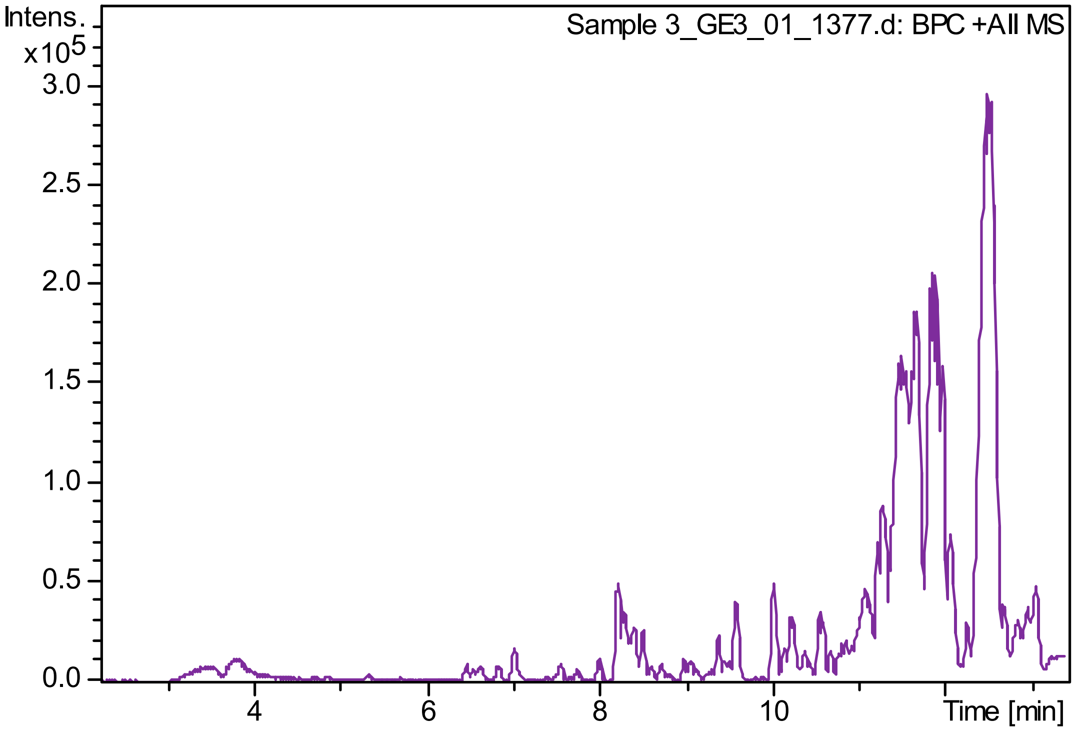

Figure S4: Total ion chromatogram of PPRF4

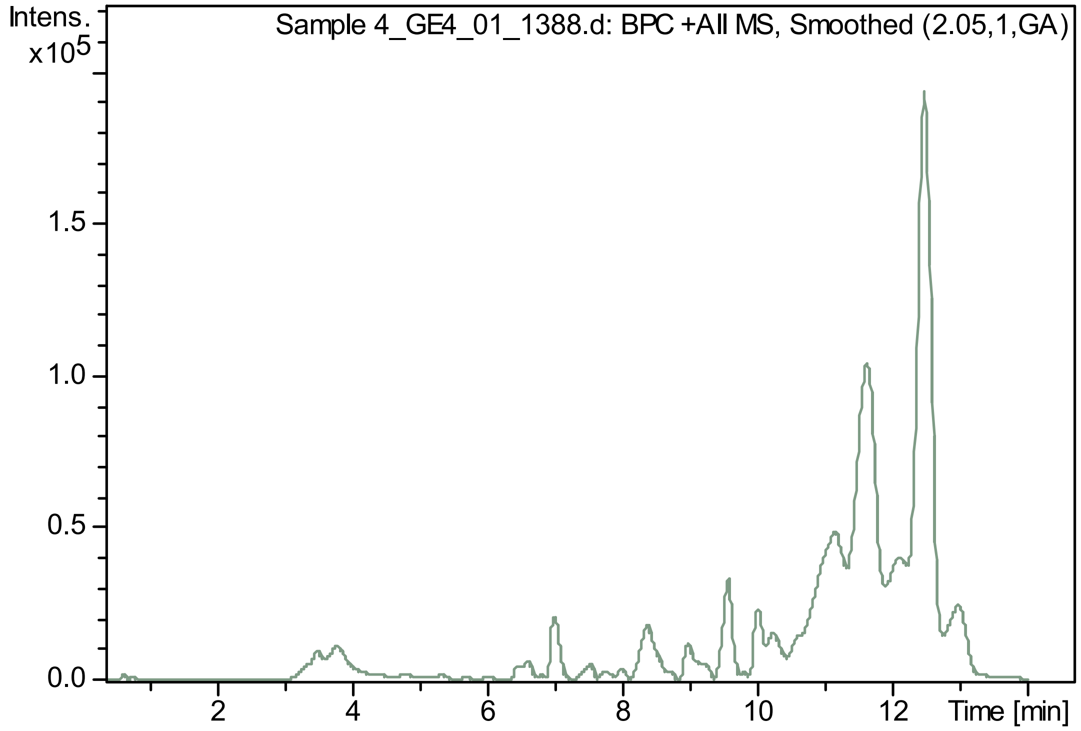

Figure S5: Total ion chromatogram of PPRF5

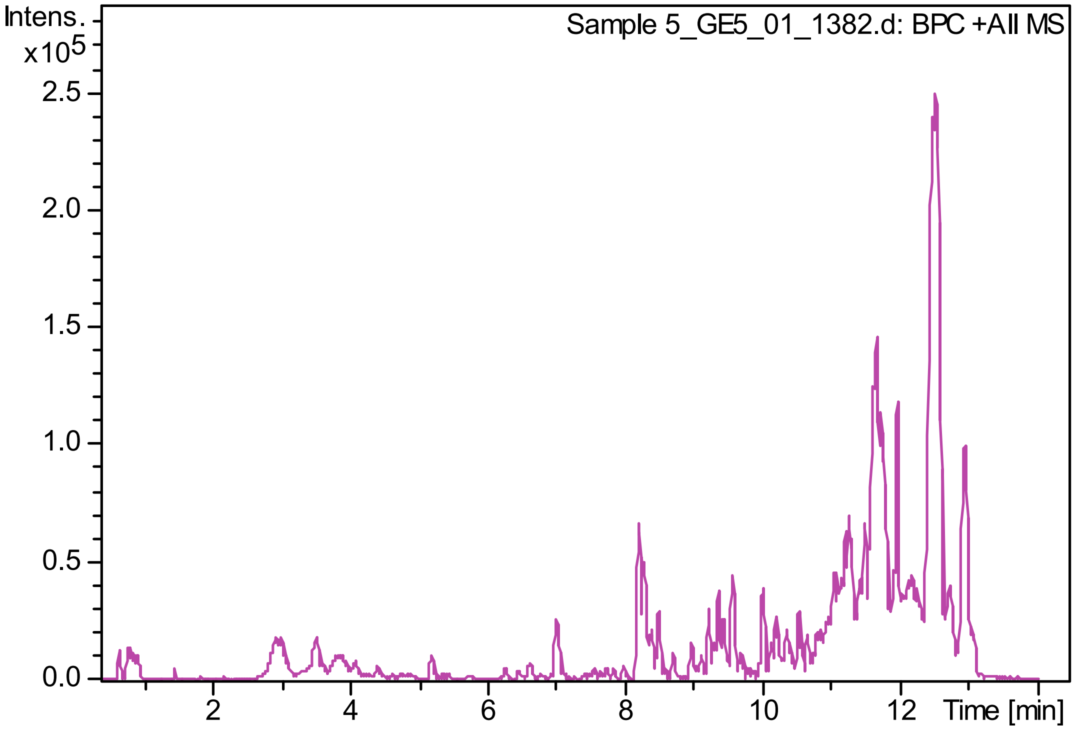

Figure S6: Total ion chromatogram of PPRFF6

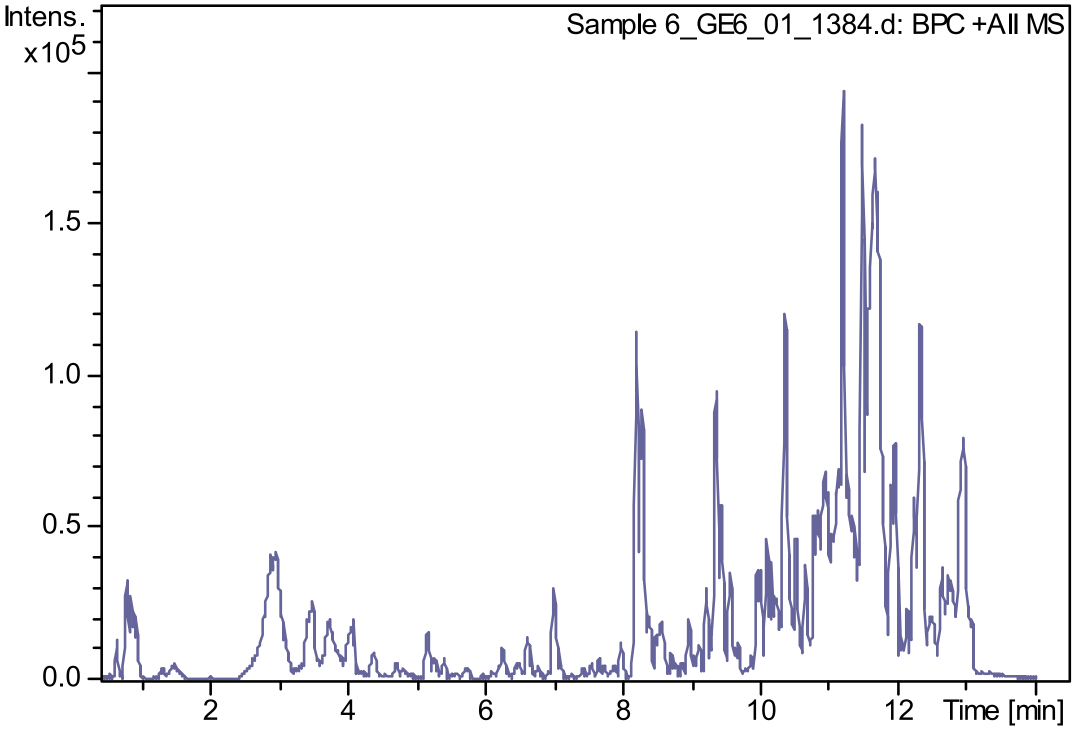

Supplement: Supplementary file 1 [file molecules-24-02118-s001.pdf]
